# GDF-15 enhances intracellular Ca^2+^ by increasing Ca_v_1.3 expression in rat cerebellar granule neurons

**DOI:** 10.1042/BCJ20160362

**Published:** 2016-06-28

**Authors:** Jun-Mei Lu, Chang-Ying Wang, Changlong Hu, Yan-Jia Fang, Yan-Ai Mei

**Affiliations:** * Institutes of Brain Science, State Key Laboratory of Medical Neurobiology and School of Life Sciences, Fudan University, Shanghai 200433, China

**Keywords:** Akt/mTOR, [Ca^2+^]_i_, Ca_v_1.3, ERK, GDF-15, TβRII

## Abstract

GDF-15 (growth/differentiation factor 15) is a novel member of the TGF (transforming growth factor)-β superfamily that has critical roles in the central and peripheral nervous systems. We reported previously that GDF-15 increased delayed rectifier outward K^+^ currents and K_v_2.1 α subunit expression through TβRII (TGF-β receptor II) to activate Src kinase and Akt/mTOR (mammalian target of rapamycin) signalling in rat CGNs (cerebellar granule neurons). In the present study, we found that treatment of CGNs with GDF-15 for 24 h increased the intracellular Ca^2+^ concentration ([Ca^2+^]_i_) in response to membrane depolarization, as determined by Ca^2+^ imaging. Whole-cell current recordings indicated that GDF-15 increased the inward Ca^2+^ current (*I*_Ca_) without altering steady-state activation of Ca^2+^ channels. Treatment with nifedipine, an inhibitor of L-type Ca^2+^ channels, abrogated GDF-15-induced increases in [Ca^2+^]_i_ and *I*_Ca_. The GDF-15-induced increase in *I*_Ca_ was mediated via up-regulation of the Ca_v_1.3 α subunit, which was attenuated by inhibiting Akt/mTOR and ERK (extracellular-signal-regulated kinase) pathways and by pharmacological inhibition of Src-mediated TβRII phosphorylation. Given that Ca_v_1.3 is not only a channel for Ca^2+^ influx, but also a transcriptional regulator, our data confirm that GDF-15 induces protein expression via TβRII and activation of a non-Smad pathway, and provide novel insight into the mechanism of GDF-15 function in neurons.

## INTRODUCTION

GDF-15 (growth/differentiation factor 15), also known as macrophage inhibitory cytokine-1, is a distant member of the TGF (transforming growth factor)-β superfamily [1]. GDF-15 plays key roles in prenatal development and the regulation of cellular responses to stress signals and inflammation as well as in tissue repair after acute injury [2]. Recent studies have also shown that GDF-15 expression is up-regulated during myocardial injury, ischaemia and remodelling, suggesting that it may act as a cytokine that protects the heart from ischaemia/reperfusion injury [3,4]. The mechanism of action of GDF-15 is not fully understood, although it was shown to block noradrenaline (norepinephrine)-induced myocardial hypertrophy by inhibiting the phosphorylation of EGFR (epidermal growth factor receptor) and the downstream kinases Akt and ERK (extracellular-signal-regulated kinase) 1/2 [5].

GDF-15 is widely expressed in the brain, specifically in the cortex, striatum and thalamus [6], and acts as a potential neurotrophic factor for midbrain dopaminergic neurons *in vivo*, promoting the survival of damaged mesencephalic dopaminergic neurons following cortical lesioning [6,7]. GDF-15 is up-regulated in a CNS (central nervous system) model of ischaemia induced by middle cerebral artery occlusion [8], and GDF-15-knockout mice exhibit progressive postnatal loss of spinal, facial and trigeminal motoneurons and sensory neurons in dorsal root ganglia [9]. An earlier study suggested that GDF-15 is involved in neuronal synaptic development and integration and may promote axonal elongation [10]. These data indicate that GDF-15 has critical roles in CNS development, although its mechanisms of action are poorly understood.

CGNs (cerebellar granule neurons) are glutamatergic cells that differentiate postnatally into various types of neuron in the mammalian brain. Primary rat CGN cultures are used as a model for studying neuronal maturation, apoptosis, differentiation and synaptic plasticity [11]. Growth and differentiation factors such as TGF-β1 and neuregulin can stimulate or inhibit CGN development and maturation via regulation of multiple signalling pathways [12,13]. GDF-15 prevented the death of K^+^-deprived CGNs by activating Akt and inhibiting constitutively active ERK [14]. We recently demonstrated that GDF-15 increased delayed rectifier outward K^+^ current (*I*_K_) and K_v_2.1 α subunit expression by Src kinase activation via TβRII (TGF-β receptor II) in non-K^+^-deprived CGN cultures [15]. These data showed for the first time that the modulation of K^+^ channel expression and the downstream signalling pathways by GDF-15 is receptor-mediated, and demonstrated that CGNs are an effective cell model for investigating the mechanism of action of GDF-15.

Increases in intracellular Ca^2+^ concentration [Ca^2+^]_i_ activate signalling pathways that induce the expression of genes essential for dendritic development, neuronal survival and synaptic plasticity [16–18]. [Ca^2+^]_i_ also regulates gene expression during CGN development [17,19]. Whether GDF-15 modulates [Ca^2+^]_i_ in CGNs and the mechanisms that are involved is unknown. In the present study, we evaluated the effect of GDF-15 on [Ca^2+^]_i_ using Ca^2+^ imaging while simultaneously recording inward Ca^2+^ current (*I*_Ca_), since changes in [Ca^2+^]_i_ in CGNs are associated with Ca^2+^ influx-dependent Ca^2+^ release [20,21]. We also examined whether the same signalling pathways and receptors identified in our previous study are activated by GDF-15 under these conditions.

## EXPERIMENTAL

### Cell culture

All experimental procedures were carried out in accordance with European guidelines for the care and use of laboratory animals (Council Directive 86/609/EEC). CGNs were derived from the cerebellum of 7-day-old Sprague–Dawley rat pups as described previously [22]. Briefly, isolated cells were plated in 35-mm-diamter Petri dishes coated with 1 μg/ml poly-L-lysine at a density of 10^6^ cells/ml and cultured at 37°C under 5% CO_2_ in DMEM (Dulbecco's modified Eagle's medium) supplemented with 10% (v/v) FBS, 5 μg/ml insulin, 25 mM KCl and 1% antibiotic/antimycotic solution. After 24 h of culture, 5 μM cytosine β-D-arabinofuranoside was added to the culture medium to inhibit the proliferation of non-neuronal cells. Cells were used for experiments after 4–5 DIC (days in culture) unless indicated otherwise.

### Patch-clamp recordings

Whole-cell CGN currents were recorded with a conventional patch-clamp technique using a multiclamp 200B amplifier (Axon Instruments) operated in voltage-clamp mode. Data acquisition and analysis were carried out using pClamp 8.01 (Axon Instruments) and/or Origin 8 (Microcal Software) software. Before recording *I*_Ca_, the culture medium was replaced with a bath solution containing 147 mM tetraethylammonium chloride, 10 mM BaCl_2_, 10 mM Hepes (pH 7.4), 2 mM MgCl_2_, 1 μM TTX (tetrodotoxin), 2 mM 4-AP (4-aminopyridine) and 10 mM glucose. Soft glass recording pipettes were filled with an internal solution containing 145 mM CsCl, 10 mM EGTA, 10 mM Hepes (pH 7.3), 5 mM Na_2_-ATP and 0.5 mM Na_2_-GTP. The pipette resistance was 4–6 MΩ after filling with internal solution. All recordings were carried out at room temperature. CGNs selected for electrophysiological recording exhibited the typical morphological characteristics of healthy cells, such as fusiform soma with two principal neurites of similar size. There was no difference in the mean capacitance of cells recorded in the control and GDF-15 treatment groups (9.17±0.23 and 9.36±0.21 pF respectively) [15].

### Western blot analysis

Cells were lysed on ice for 30 min in lysis buffer containing 20 mM Hepes, 150 mM NaCl, 0.5% Nonidet P-40, 10% glycerol, 2 mM EDTA, 100 μM Na_3_VO_4_, 50 mM NaF (pH 7.5) and 1% proteinase inhibitor cocktail. After centrifugation, the supernatant was mixed with 2× SDS loading buffer and boiled for 5 min. Proteins were separated by SDS/PAGE (10% gel) and transferred on to a PVDF membrane (Millipore), which was blocked with 10% (w/v) non-fat dried skimmed milk powder and incubated at 4°C overnight with mouse monoclonal antibody against Ca_v_1.2 or Ca_v_1.3 (1:1000 dilution; NIH NeuroMab Facility/University of California Davis) and mouse monoclonal antibody against GAPDH (glyceraldehyde-3-phosphate dehydrogenase) (1:10000 dilution; KangChen Bio-Tech). After extensive washing in TBS with 0.1% Tween 20, the membrane was incubated with horseradish peroxidase-conjugated anti-mouse or anti-rabbit IgG (1:10000 dilution; KangChen Bio-Tech) for 2 h at room temperature. Protein bands were visualized by chemiluminescence using the SuperSignal West Pico trial kit (Pierce) and detected using a ChemiDoc XRS system (Bio-Rad Laboratories). Quantity One version 4.6.2 software (Bio-Rad Laboratories) was used for background subtraction and quantification of immunoblotting data.

### Measurement of [Ca^2+^]_i_

[Ca^2+^]_i_ in single cells was detected on the basis of fura 2 fluorescence intensity as described previously [23]. Briefly, CGNs grown on coverslips were rinsed twice with BSS (balanced salt solution) containing 145 mM NaCl, 2.5 mM KCl, 10 mM Hepes, 1 mM MgCl_2_, 10 mM glucose and 2 mM CaCl_2_, and incubated at 37°C for 45 min in the presence of fura-2 AM (fura 2 acetoxymethyl ester) with 0.1% DMSO in BSS. After two washes with BSS, cells were incubated for an additional 20 min in BSS before imaging. The coverslips were transferred to a chamber mounted on the stage of an inverted phase-contrast microscope (Nikon Eclipse Ti); fresh BSS was added to the chamber, and images were acquired at 4-s intervals for the duration of the experiment. Excitation wavelengths for fura 2 were 340 and 380 nm, with emission at 505 nm. Baseline [Ca^2+^]_i_ was determined for 60 s immediately before the addition of high-K^+^ solution (27 mM KCl). Fluorescence intensity was quantified using Metafluor software (Universal Imaging Corporation).

### Transfection and dual luciferase reporter assays

Rat Ca_v_1.3 promoter (−1400 to +497 bp) synthesized by Magorbio was inserted into pGL3 luciferase reporter plasmid. Lentiviral vectors for co-ordinately expressing *CACNA1D* (L-type voltage-dependent Ca^2+^ channel α1D subunit) promoter following fluorescent protein and *Renilla* luciferase protein only were constructed respectively. CGNs cells were co-transfected with Ca_v_1.3 promoter and *Renilla* reporter plasmids. Luciferase assays were performed at 7 DIC which is 3 days after transfection using the Dual Luciferase Reporter Assay system (Promega) according to the manufacturer's instructions. The results were expressed as a ratio of firefly luciferase (Fluc) activity to *Renilla* luciferase (Rluc) activity, and the *Renilla* luciferase reporter gene (50 ng) was used as an internal control. For each sample, the relative luciferase activity was normalized to the control group Fluc/Rluc ratio. All experiments were performed in triplicate.

### qPCR

To measure the Ca_v_1.2 and Ca_v_1.3 mRNA levels, qPCR (quantitative real-time PCR) analysis was performed with the following sequences: Ca_v_1.2 forward primer 5′-TCAAAGGCTACCTGGACTGGAT-3′ and reverse primer 5′-CCATGCCCTCG TCCTCATT-3′; Ca_v_1.3 forward primer 5′-CTTCCTCTTCATCATCATCTTC-3′ and reverse primer 5′-TCATACATCACCGCATTCC-3′. To control for sampling errors, qPCR for the housekeeping gene *GAPDH* was performed with the primer sequences 5′-TGCTCCTCCCTGTTC-3′ (forward) and 5′-AGCCTTGACTGTGCC-3′ (reverse). The reaction solution contained 1.0 μg of diluted reverse transcription PCR product, 0.2 μM of each paired primer and Power SYBR Green PCR master mix (Toyobo). The annealing temperature was set at 58°C and 40 amplification cycles were used. The absolute mRNA levels in each sample were calculated according to a standard curve determined using serial dilutions of known amounts of specific templates plotted against the corresponding cycle threshold (*C*_T_) values. The normalized ratio of the target gene over *GAPDH* in each sample was calculated. The specificity of the primers was verified by both gel electrophoresis and sequencing of the PCR products.

### Data analysis

Multiple groups were compared by one-way ANOVA and two-sample comparisons were performed using Student's *t* test. Results are presented as means±S.E.M., with *n* as the number of neurons recorded, imaging experiments or replicates. Electrophysiological data were collected from at least four different batches of neurons prepared on different days to minimize bias resulting from culture conditions. *P*<0.05 was considered statistically significant.

### Chemicals

Recombinant human GDF-15 was purchased from Pepro Tech. TTX, 4-AP, rapamycin, SB431542, PP1, LY2109761 and poly-L-lysine were purchased from Sigma. U0126 was purchased from Selleckchem. FBS, DMEM and antibiotic/antimycotic solution were purchased from Gibco Life Technologies.

## RESULTS

### GDF-15 enhances [Ca^2+^]_i_ and *I*_Ca_ in CGNs without affecting steady-state channel activation

**Figure 1:**
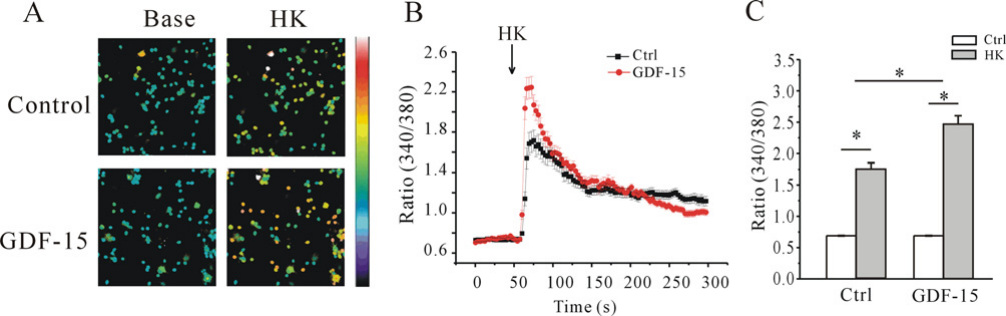
Effect of GDF-15 on [Ca^2+^]_i_ induced by high K^+^ in rat CGNs (**A**) Intracellular Ca^2+^ imaging of control and GDF-15-treated CGNs before (Base) and after (HK) depolarization by acute perfusion with 27 mM K^+^. Changes in fura 2 fluorescence excitation ratios with increasing [Ca^2+^]_i_ are depicted as a colour gradient from purple to red. Scale bar, 50 μm. (**B**) Changes in [Ca^2+^]_i_ upon application of a depolarizing stimulus, as measured by quantification of fluorescence excitation ratios. The arrow represents a 30-s perfusion with a depolarizing solution of 27 mM K^+^. (**C**) Statistical analysis of [Ca^2+^]_i_ induced by high K^+^ in the presence or absence of GDF-15. Results are means±S.E.M. *P<0.05 for the two groups connected with a straight line. Ctrl, control.

We demonstrated previously that GDF-15 increases *I*_K_ of CGNs in a time- and dose-dependent manner at different developmental stages, and that incubating CGNs starting from 5 DIC with 100 ng/ml GDF-15 for 24 h produced the most significant increase in *I*_K_ [15]. We therefore applied 100 ng/ml GDF-15 to CGNs after 5 DIC for 24 h and evaluated the effects of GDF-15 on [Ca^2+^]_i_ by Ca^2+^ imaging using the Ca^2+^-sensitive fluorescent dye fura 2. Since GDF-15 did not affect basal [Ca^2+^]_i_, we used a high-K^+^ solution (27 mM KCl) to depolarize neurons and activate VGCCs (voltage-gated Ca^2+^ channels), inducing a rapid increase in [Ca^2+^]_i_. In control neurons, depolarization with high K^+^ caused acute elevation of [Ca^2+^]_i_, with an increase in the *F*_340_/*F*_380_ ratio from 0.69±0.01 (*n*=43) to a maximum of 1.71±0.06 (*n*=127). After treatment with GDF-15 for 24 h, the ratio increased to a maximum value of 2.24±0.01 (*n*=115) (Figures 1A and 1B), which was ∼30.9% higher than in the control (Figure 1C).

**Figure 2:**
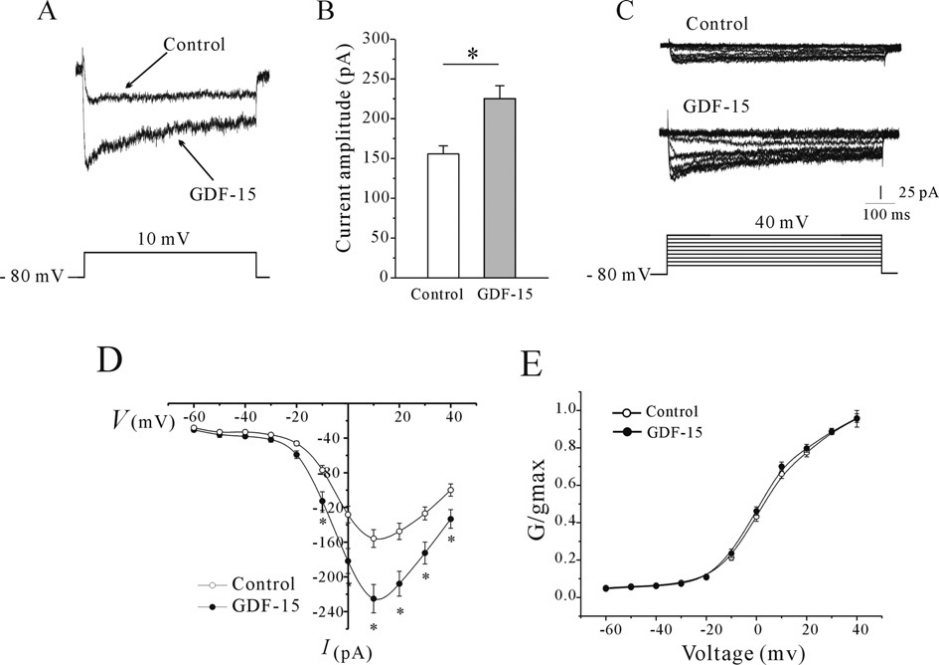
Effect of GDF-15 on *I*_Ca_ amplitude and steady-state Ca^2+^ channel activation (**A**) Representative traces of control and GDF-15-treated CGNs. *I*_Ca_ was elicited by depolarization to 10 mV from a holding potential of −80 mV. (**B**) Statistical analysis of the effect of GDF-15 on *I*_Ca_ amplitude. Results are means±S.E.M. *P<0.05 for two groups connected with a straight line. (**C**) Representative traces obtained with a steady-state voltage protocol of control and GDF-15-treated CGNs. *I*_Ca_ was elicited by 200-ms depolarizing pulses from a holding potential of −80 mV to between −60 and +40 mV in 10-mV steps at 10-s intervals. (**D**) Voltage-dependent activation curves of *I*_Ca_. *P<0.05 compared with corresponding control. (**E**) Steady-state activation curves of *I*_Ca_ obtained by plotting normalized conductance as a function of command potential. Data points were fitted using the Boltzmann function. Results are means±S.E.M.

To assess the role of GDF-15 in the activation of VGCCs in CGNs, we recorded whole-cell *I*_Ca_, which was evoked by a 200-ms depolarization from a holding potential of −80 to 10 mV. GDF-15 application (100 ng/ml for 24 h) increased the *I*_Ca_ amplitude by 44.57% (from 155.71±10.23 to 225.11±16.31 pA, *n*=41 and 54, *P*<0.05) (Figures 2A and 2B). We then investigated whether the effects of GDF-15 on the *I*_Ca_ amplitude were exerted via modulation of the voltage-gating properties of *I*_Ca_ channels. An *I*_Ca_ was evoked by a 20-ms depolarizing pulse from a holding potential of −80 mV to between −60 and 40 mV in 10-mV steps at 10-s intervals (Figure 2C). The current–voltage (*I*–*V*) curves of control and GDF-15-treated CGNs showed that *I*_Ca_ increased from a negative potential of −40 mV to a maximum value of 10 mV (Figure 2D), indicating that GDF-15 did not affect Ca^2+^ channel activity. Steady-state *I*_Ca_ activation was determined by calculating conductance and normalizing this value to the command voltage. Data were fitted using the Boltzmann function. The steady-state *I*_Ca_ activation curves of CGNs with or without GDF-15 treatment showed half-activation potentials of 4.57±1.36 and 3.24±1.14 mV respectively (*n*=15 and 18 respectively; *P* > 0.05) (Figure 2E). These results indicate that GDF-15-induced increases in *I*_Ca_ amplitude were not due to changes in the voltage-gating properties of Ca^2+^ channels.

### L-type Ca^2+^ channels and Ca_v_1.3 expression mediate the GDF-15-induced [Ca^2+^]_i_ and *I*_Ca_ amplitude

**Figure 3:**
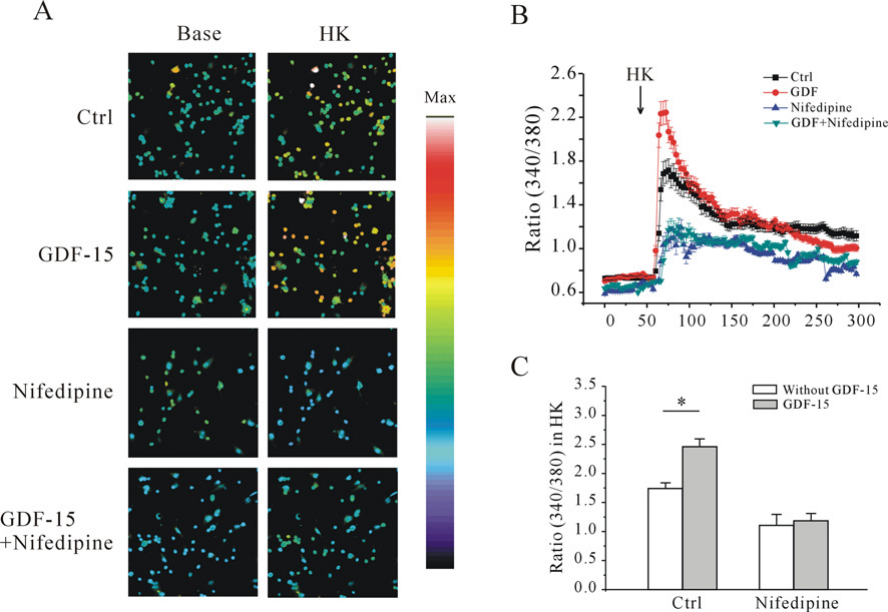
Effect of nifedipine on the increase in [Ca^2+^]_i_ elicited by high K^+^ in CGNs with or without GDF-15 treatment (**A**) Ca^2+^ imaging before and after depolarization by application of a 27-mM K^+^ solution in GDF-15-treated CGNs in the presence or absence of nifedipine. Scale bar, 50 μm. (**B**) Changes in [Ca^2+^]_i_ upon application of a depolarizing stimulus, as measured by quantification of fluorescence excitation ratios. Each arrow represents a 30-s perfusion with a depolarizing 27-mM K^+^ solution. (**C**) Statistical analysis of [Ca^2+^]_i_ in control and GDF-15-treated CGNs in the presence or absence of nifedipine. Results are means±S.E.M. *P<0.05 for the two groups connected with a straight line. Ctrl, control.

**Figure 4:**
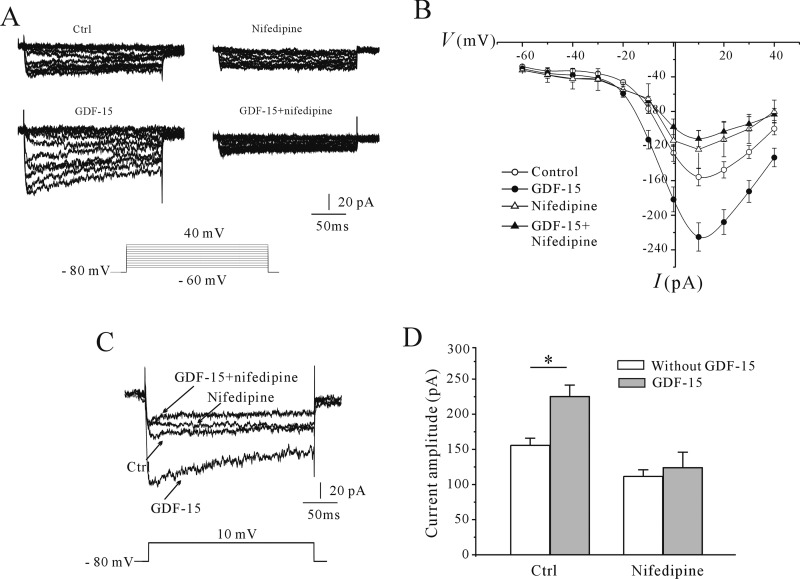
Effect of nifedipine on *I*_Ca_ in control and GDF-15-treated CGNs (**A**) Representative traces obtained with a steady-state voltage protocol of control and GDF-15-treated CGNs in the presence or absence of nifedipine. (**B**) *I*–*V* curve of *I*_Ca_. Results were obtained from six independent experiments and are means±S.E.M. *P<0.05. (**C**) Representative traces of control and GDF-15-treated CGNs in the presence or absence of nifedipine. *I*_Ca_ was elicited with depolarizing pulses to 10 mV from a holding potential of −80 mV. (**D**) Statistical analysis of the effect of nifedipine on *I*_Ca_. Results are means±S.E.M. *P<0.05 for the two groups connected with a straight line. Ctrl, control.

The *I*–*V* curves suggested that the Ca^2+^ channels were L-type channels found in neurons [24]. To determine whether L-type Ca^2+^ channels are indeed responsible for the GDF-15-induced increases in Ca^2+^ influx and *I*_Ca_ amplitude, we treated CGNs with the selective blocker nifedipine. Pre-incubation of CGNs with nifedipine (10 μM) [25] abrogated the increase in [Ca^2+^]_i_ evoked by high K^+^ and inhibited the GDF-15-induced increase in [Ca^2+^]_i_ (Figures 3A and 3B). In the presence of nifedipine, the increase in the *F*_340_/*F*_380_ ratio evoked by high-K^+^ solution without and with GDF-15 was reduced from 1.71±0.06 (*n*=127) to 1.15±0.10 (*n*=58) and from 2.24±0.01 (*n*=115) to 1.23±0.07 (*n*=45) respectively (Figure 3C). Consistent with these findings, nifedipine application alone reduced the amplitude of *I*_Ca_ evoked by a 200-ms depolarization from −80 to 10 mV by 29.95±5.9% (*n*=24 and 7), and abolished the GDF-15-induced increase in *I*_Ca_ amplitude (Figures 4A and 4B), suggesting that L-type Ca^2+^ channels mediate the GDF-15-induced increase in the *I*_Ca_ amplitude and [Ca^2+^]_i_.

**Figure 5:**
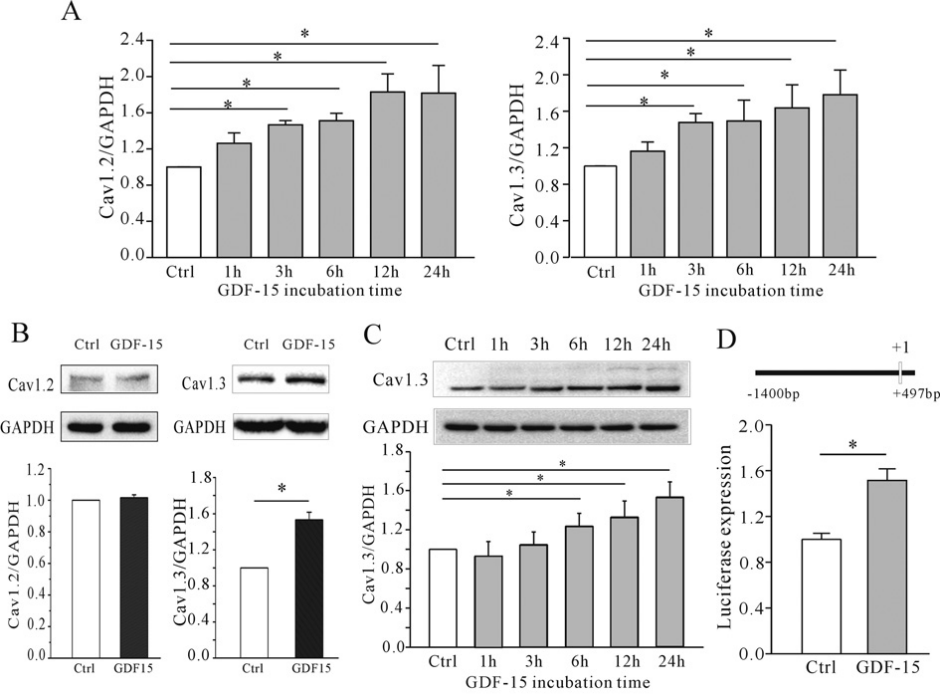
Effect of GDF-15 on Ca_v_1.2 and Ca_v_1.3 expression in CGNs (**A**) Statistical analyses of Ca_v_1.2 and Ca_v_1.3 mRNA levels detected using qPCR. CGNs were incubated with GDF-15 from 15 min to 36 h. (**B**) Western blot and statistical analyses of the effect of GDF-15 on Ca_v_1.2 and Ca_v_1.3 expression in CGNs. (**C**) Western blot and statistical analyses of Ca_v_1.3 levels in CGNs after incubation with GDF-15 for 15 mins to 36 hrs. (**D**) Statistical analyses of the effect of GDF-15 on Ca_v_1.3 promoter expression in CGNs determined by luciferase reporter assays. Promoter information is illustrated. Results are means±S.E.M. *P<0.05 for the two groups connected with a straight line. Ctrl, control.

We investigated whether the GDF-15-mediated increase in *I*_Ca_ is due to an up-regulation of channel expression. A previous study showed that Ca_v_1.2 and Ca_v_1.3 are the major α-subunits of L-type Ca^2+^ channels [26]; we therefore assessed the expression of these two proteins in GDF-15-treated cells. Specific primers to amplify Ca_v_1.2 and Ca_v_1.3 were used to measure mRNA expression levels by qPCR after incubation with and without GDF-15. The results reveal that there was a significant increase in the mRNA levels both of the Ca_v_1.2 and Ca_v_1.3 α-subunit (Figure 5A). However, Western blotting indicated that only the Ca_v_1.3 but not the Ca_v_1.2 protein level was increased in CGNs by 53.34±8.46% (*n*=4; *P*<0.05) following 24 h of incubation with GDF-15 (100 ng/ml) at 5 DIC (Figure 5B). Moreover, the effect of GDF-15 on Ca_v_1.3 expression was gradually increased with incubation time (Figure 5C). These results suggest that an up-regulation in Ca_v_1.3 protein expression induced by GDF-15 is responsible for the observed increased in *I*_Ca_ and [Ca^2+^]_i_. We thus examined the effect of GDF-15 on the Ca_v_1.3 gene promoter using luciferase reporter assays. Administration of GDF-15 increased luciferase expression driven by the rat Ca_v_1.3 promoter by 51.4±17.6% (*n*=3; Figure 5D).

### Effect of GDF-15 on Ca_v_1.3 expression requires Akt/mTOR and MAPK (mitogen-activated protein kinase)/ERK activation via TβRII

**Figure 6:**
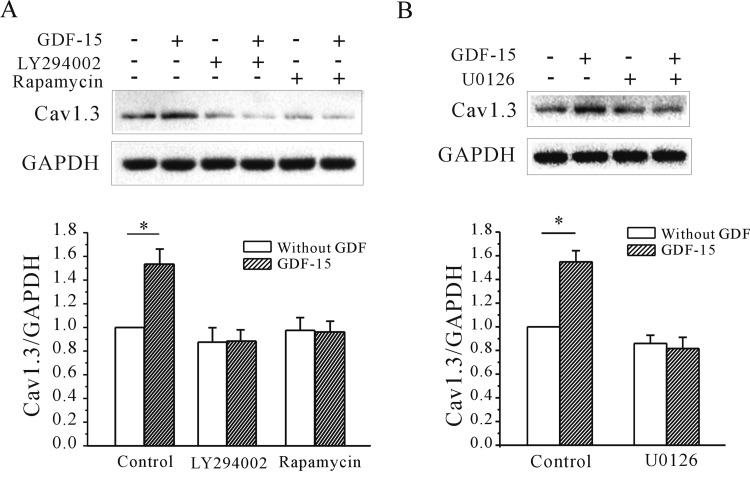
Effect of Akt/mTOR and ERK pathway inhibition on the GDF-15-induced increase in Ca_v_1.3 α subunit expression (**A** and **B**) Western blot and statistical analyses of the effects of the Akt inhibitor LY294002 and mTOR inhibitor rapamycin (**A**) and the MEK inhibitor U0126 (**B**) on GDF-15-induced up-regulation of Ca_v_1.3 protein levels. Results are means±S.E.M. *P<0.05 for the two groups connected with a straight line.

Our previous study showed that Akt/mTOR signalling and TβRII activity are required for the GDF-15-induced up-regulation of *I*_K_ and K_v_2.1 α subunit expression [15]. We therefore investigated whether these are involved in the observed effect of GDF-15 on Ca_v_1.3 expression. Blocking Akt/mTOR activity with 20 μM LY294002 or 50 nM rapamycin [27] reduced the GDF-15-induced increase in Ca_v_1.3 protein expression from 53.4±6.7% to 11.6±4.7% and 0.04±4.2%, respectively (*n*=3; *P*<0.05) (Figure 6A). Unexpectedly, inhibition of MAPK with 1 μM U0126 [28] also suppressed the increase in Ca_v_1.3 expression induced by GDF-15 from 54.8±4.7% to 18.4±4.3% (*n*=3, *P*<0.05) (Figure 6B). These data indicate that both the Akt/mTOR and MAPK/ERK pathways are required for the up-regulation of Ca_v_1.3 expression induced by GDF-15.

**Figure 7:**
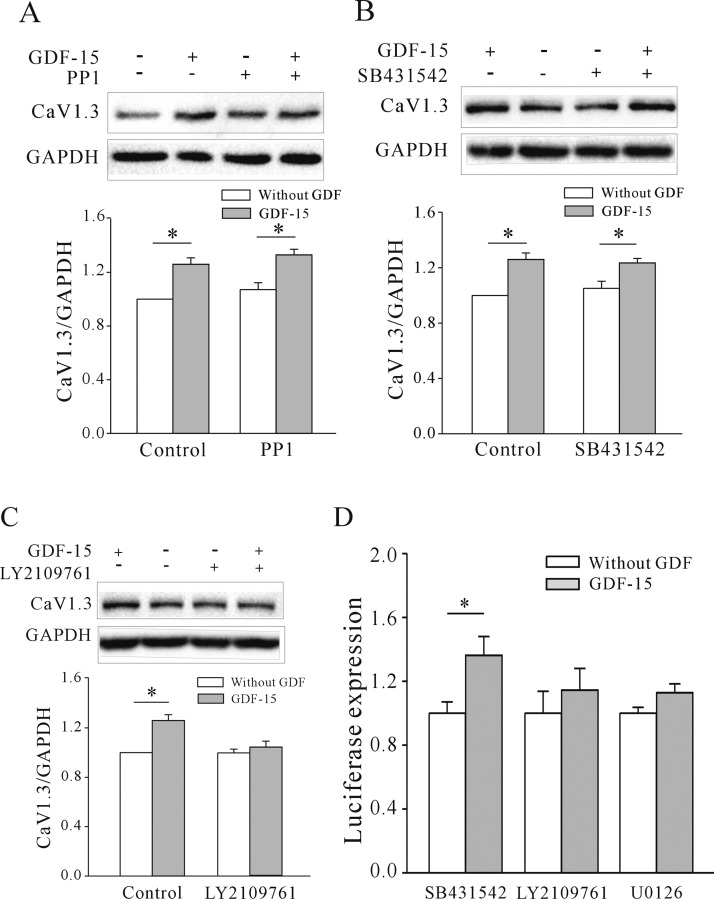
Effects of TβRI and TβRI/TβRII inhibitors on the GDF-15-induced increase in Ca_v_1.3 protein level and gene promoter expression (**A**–**C**) Western blot and statistical analyses of the effects of TβRI inhibitors (PP1 and SB431542) (**A** and **B**) and TβRI/TβRII dual inhibitor (LY2109761) (**C**) on GDF-15-induced up-regulation of Ca_v_1.3 protein levels. (**D**) Statistical analyses of the effect of the effects of SB431542, LY2109761 and U0126 on GDF-15-induced up-regulation of Ca_v_1.3 promoter expression in CGNs determined by luciferase reporter assays. Results are means±S.E.M. *P<0.05 for the two groups connected with a straight line.

Since there are no specific inhibitors of TβRII, we used the TβRI inhibitors SB431542 and PP1 and the TβRI/TβRII inhibitor LY2109761 to determine whether the effect of GDF-15 on Ca_v_1.3 expression involves TβRII. There was no change in GDF-15-induced Ca_v_1.3 expression relative to the control upon treatment with 10 μM PP1 [15] (29.5±3.91% without PP1 compared with 34.11±4.94% with PP1; *n*=5, *P* > 0.05) (Figure 7A) or 10 μM SB431542 [15] (29.5±3.91% without SB431542 compared with 25.22±3.41% with SB431542, *n*=5, *P* > 0.05) (Figure 7B). In contrast, 5 μM LY2109761 [15] treatment reduced the GDF-15-induced up-regulation of Ca_v_1.3 expression from 29.5±3.91% to 1.41±4.99% (*n*=5; *P*<0.05) (Figure 7C). These results indicate that the effects of GDF-15 on *I*_Ca_ and [Ca^2+^]_i_ are exerted via modulation of Ca_v_1.3 expression, which involves the activation of Akt/mTOR and MAPK/ERK signalling downstream of TβRII. Furthermore, we also examined whether TβRII and ERK signal pathways are involved in the observed effect of GDF-15 on the Ca_v_1.3 gene promoter using luciferase reporter assays. Similarly, together with SB431542, administration of GDF-15 significantly increased luciferase expression driven by the rat Ca_v_1.3 promoter by 36.23±11.82% relative to the control upon treatment with SB431542 alone. LY2109761 and U0126 inhibited the GDF-15-induced up-regulation of luciferase expression driven by the rat Ca_v_1.3 promoter (*n*=3, Figure 7D).

## DISCUSSION

GDF-15 plays various roles in neuroprotection, neural regeneration and axonal elongation [6,8,9]. However, there is little known about the mechanism of action of GDF-15 and its downstream effectors. Our previous study suggested that GDF-15 activates TβRII and PI3K (phosphoinositide 3-kinase)/Akt/mTOR signalling to increase the *I*_K_ amplitude and K_v_2.1 expression in CGNs, which may have developmental significance [15]. In the present study, we found that GDF-15 also increased the expression of Ca_v_1.3 and thereby modulated the *I*_Ca_ and [Ca^2+^]_i_, which involved activation of the same receptor and some of the same downstream signalling components as those previously reported by our group.

VGCCs are voltage sensors that convert membrane depolarization into intracellular Ca^2+^ signals. In neurons, VGCCs are L-, N-, P/Q-, R- and T-type Ca^2+^ channels [16,24]. L-type Ca^2+^ channels are widely distributed on the neuronal cell body throughout the mammalian CNS, including in CGNs [26,29]. Ca^2+^ influx in response to membrane depolarization occurs via L-type Ca^2+^ channels and regulates intracellular Ca^2+^ homoeostasis [18,30]. Our results demonstrate that intracellular basal Ca^2+^ was not increased by GDF-15 treatment; however, [Ca^2+^]_i_ in response to membrane depolarization and nifedipine-sensitive *I*_Ca_ were up-regulated, suggesting the involvement of L-type Ca^2+^ channels, although we cannot exclude the possibility that nifedipine-insensitive Ca^2+^ channels or N-, P/Q- or R-type Ca^2+^ channels were also modulated by GDF-15. A study of rat CGNs indicated that administration of PACAP (pituitary adenylate cyclase-activating polypeptide) induced a rapid rise in [Ca^2+^]_i_ and thereby stimulated Ca^2+^ influx through N-type but not L-type Ca^2+^ channels [31]. This difference may be explained by the fact that PACAP affects basal [Ca^2+^]_i_, but not the response to membrane depolarization. Moreover, PACAP modulated the channels through rapid phosphorylation of channel proteins rather than regulation of Ca^2+^ channel α subunit expression [31].

L-type channels consist of subtypes Ca_v_1.1–Ca_v_1.4. Ca_v_1.1 and Ca_v_1.4 are mainly expressed in skeletal muscle and retinal cells [32,33], whereas Ca_v_1.2 and Ca_v_1.3 are abundant in the brain [29,34]. Both of the latter isoforms show broad expression patterns in many types of neuron [35,36], where they regulate neuronal excitability, synaptic plasticity and activity-dependent gene transcription [37–39]. Ca_v_1.2 and Ca_v_1.3 account for 89% and 11% of L-type channel transcripts respectively in mouse CGNs, and Ca_v_1.2 comprises the pore-forming subunits of anomalous L-type channels in these cells [40]. However, our data showed that there was no difference in the expression of the two isoforms in CGNs, consistent with a previous study reporting that functionally distinct L-type Ca^2+^ channels coexist in rat CGNs [41]. Besides species differences, variations in protein stability probably underlie the higher abundance of the Ca_v_1.3 α subunit than what is predicted from mRNA levels, leading to a higher number of functional Ca_v_1.3 channels in the membrane.

Ca_v_1.3 and Ca_v_1.2 differ in terms of biophysical properties, distribution in the brain and function [42,43]. We observed that the expression of the two channel types is also differentially regulated, since GDF-15 up-regulated the expression of Ca_v_1.3 protein but not Ca_v_1.2 protein, but both Ca_v_1.2 and Ca_v_1.3 mRNA levels detected by using quantitative real-time PCR were increased by GDF-15 for reasons that are unclear. Ca_v_1.2 and Ca_v_1.3 are encoded by the *cacna1C* and *cacna1D* genes respectively [44]. The regulatory properties of Ca_v_1.2 and Ca_v_1.3 channels differ according to interaction with different intracellular proteins [45,46]. For instance, the association between Ca_v_1.2 and PDZ (PSD-95/Dlg/ZO1) domain proteins plays an important role in coupling L-type Ca^2+^ channel activity with the phosphorylation of nuclear CREB (cAMP-response-element-binding protein) [47], whereas interaction of Ca_v_1.3 with Shank results in its targeting to phosphorylated (p)CREB at synapses [45,48]. Structurally distinct forms of Ca_v_1.3 have also been reported in which the C-terminal modulatory domain confers unique gating properties [49,50]. Whether the differential regulation of Ca_v_1.2 and Ca_v_1.3 protein expression by GDF-15 is due to variation in protein structure or a post-transcriptional mechanism remains an open question.

Our previous study found that Akt/mTOR and MAPK/ERK pathways were activated in CGNs by GDF-15 treatment, consistent with findings in non-neuronal cells [51,52], although activation of ERK signalling was not required for the GDF-15-induced increases in K_v_2.1 expression and *I*_K_ [15]. Moreover, the effect of GDF-15 on K_v_2.1 expression may be exerted via TβRII-induced activation of Src [15]. The results of the present study suggest that the up-regulation of Ca_v_1.3 expression induced by GDF-15 is required for the activation of TβRII and PI3K/Akt/mTOR signalling pathways, confirming our previous finding of a non-Smad mechanism [15]. However, we observed that blocking ERK signalling did abolish the GDF-15-induced increase in Ca_v_1.3 expression, suggesting that activation of the ERK pathway is required for this effect. A previous study showed that ERK activation regulates K_v_4 channel subunits at the transcriptional and post-translational levels [53]. ERK can directly phosphorylate ion channel subunits and may alter the gating properties of K^+^ channels, as in the regulation of *I*_K_ by growth factors [54]. Since GDF-15 neither alters the gating properties of *I*_Ca_ nor has an immediate effect on Ca^2+^ amplitude (results not shown), instead it increased the mRNA level of Ca_v_1.3 and luciferase expression driven by Ca_v_1.3 promoter, we believe that the activation of ERK signalling by GDF-15 regulates Ca_v_1.3 expression at the transcriptional level.

Neuronal L-type Ca^2+^ was known to play a critical role in coupling neuronal activity to gene transcription. Ca^2+^ influx via postsynaptic L-type Ca^2+^ channels activates pCREB [47,48] and NFATc4 (nuclear factor of activated T-cells cytoplasmic 4) [55], which stimulate the transcription of target genes [48]. However, a recent study demonstrated that the C-terminus of Ca_v_1.3 translocates to the nucleus where it functions as a transcriptional regulator to modulate the transcription of Ca^2+^-activated K^+^ channels in atrial myocytes [56], and studies in Ca_v_1.3^−/−^ mice have implicated Ca_v_1.3 channels in auditory brainstem physiology and development [57,58]. Various pathologies have been linked to Ca_v_1.3 channels; for instance, Ca_v_1.3 channel deficiency reduces long-term fear memory, antidepressant-like behaviour and congenital deafness [59–61]. In conclusion, our findings provide important insight into the mechanisms underlying the various functions of GDF-15 in the brain.

## Abbreviations

4-AP: 4-aminopyridine
BSS: balanced salt solution
[Ca^2+^]_i_: intracellular Ca^2+^ concentration
CGN: cerebellar granule neuron
CNS: central nervous system
CREB: cAMP-response-element-binding protein
DIC: days in culture
DMEM: Dulbecco's modified Eagle's medium
ERK: extracellular-signal-regulated kinase
Fluc: firefly luciferase
GAPDH: glyceraldehyde-3-phosphate dehydrogenase
GDF-15: growth/differentiation factor 15
*I*_Ca_: inward Ca^2+^ current
*I*_K_: delayed rectifier outward K^+^ current
MAPK: mitogen-activated protein kinase
PACAP: pituitary adenylate cyclase-activating polypeptide
PI3K: phosphoinositide 3-kinase
qPCR: quantitative real-time PCR
Rluc: *Renilla* luciferase
TβR: TGF-β receptor
TGF: transforming growth factor
TTX: tetrodotoxin
VGCC: voltage-gated Ca^2+^ channel
